# Antimicrobial, Anthelmintic Activities and Characterisation of Functional Phenolic Acids of *Achyranthes aspera* Linn.: A Medicinal Plant Used for the Treatment of Wounds and Ringworm in East Africa

**DOI:** 10.3389/fphar.2015.00274

**Published:** 2015-11-23

**Authors:** Ashwell R. Ndhlala, Habteab M. Ghebrehiwot, Bhekumthetho Ncube, Adeyemi O. Aremu, Jiří Gruz, Michaela Šubrtová, Karel Doležal, Christian P. du Plooy, Hafiz A. Abdelgadir, Johannes Van Staden

**Affiliations:** ^1^Research Centre for Plant Growth and Development, School of Life Sciences, University of KwaZulu-NatalPietermaritzburg, South Africa; ^2^Vegetable and Ornamental Plants, Agricultural Research CouncilPretoria, South Africa; ^3^African Centre for Crop Improvement, University of KwaZulu-NatalPietermaritzburg, South Africa; ^4^Department of Chemical Biology and Genetics, Centre of the Region Haná for Biotechnological and Agricultural Research, Faculty of Science, Palackỳ University and Institute of Experimental Botany, Academy of Sciences of Czech RepublicOlomouc-Holice, Czech Republic

**Keywords:** Amaranthaceae, chlorogenic acid, genistein, protein binding, rutin, taxifolin, wound healing

## Abstract

*Achyranthes aspera* Linn. (Amaranthaceae) commonly known as Prickly Chaff flower (English) is traditionally used for treating a number of ailments. Different parts of the plant are used in treating wounds and ringworm in East Africa and elsewhere for a number of ailments. In this study, leaf extracts of *A. aspera* collected from two different geographical locations (Ciaat, Eritrea and Ukulinga, South Africa) were evaluated for antibacterial, antifungal, anthelmintic activities and the plant characterized for functional phenolic acids as well as protein binding capacity. The pathogens used in the tests were, two Gram-negative (*Escherichia coli* and *Klebsiella pneumoniae*), two Gram-positive bacteria (*Bacillus subtilis* and *Staphylococcus aureus*), a filamentus yeast-like fungus (*Candida albicans*) and a free-living nematode (*Caenorhabditis elegans*). The water and acetone extracts of the samples collected from Ciaat exhibited good antibacterial, antifungal and anthelmintic activity (MIC < 1 mg/ml) except the water extract against *E. coli* which showed moderate activity. In contrast, the extracts collected from Ukulinga exhibited moderate to weak activities except for the acetone (aq.) extracts which had good activity against some of the tested organisms. UHPLC-MS/MS revealed variation in the levels of some functional phenolic compounds, with rutin, chlorogenic acid and genistein not being detected in the extracts from Ukulinga. The variation was also observed in the protein binding capacity, which could offer a predictive wound healing model. All extracts from plant samples collected at Ciaat expressed significant dominant potency compared to similar extracts from Ukulinga.

## Introduction

Throughout the history of traditional medical systems, plants have always been used as ingredients in most of the treatment therapies. Plants synthesize many diverse chemical molecules which, although perceived as waste and detoxification products or expression of shunt and overflow metabolism, continue to benefit humankind (Ncube and Van Staden, 2015). The use of plants in traditional health care systems is thus rooted on the exploitation of this biogenic pool of metabolites.

Although, traditional medicinal systems hinges on human cultures, their persistence is, however, rooted on the ability of individuals to learn from and copy each other through cultural transmission (Cavalli-Sforza et al., 1982; Pennisi, 2010). The evolutionary success of these cultural traits derives this credit from their constant and cumulative natural selection over generations, an attribute that allows waves of innovations and trait modifications to become continuously assimilated (Tennie et al., 2009). Ethnopharmacology, thus serves as a multidisciplinary approach and field of inquiry that investigate the anthropological rationale and pharmacological basis of the medicinal plants used by diverse human cultures. The ethnopharmacological approaches have provided major innovations and breakthroughs toward the understanding and appraisal of traditional medicine and its accelerated integration into mainstream medicine. To this end, one may argue on the precise percentage of the world’s population that use traditional medicines. Irrespective of what the different reasons are for using it, there is no doubt a significant portion which rely on or choose it consciously as an alternative to mainstream medicine. In Africa, for example, WHO (2008) estimates this figure to be 70% of the total African population.

In addition to its heterogeneity and holistic approach to the treatment of various ailments, traditional medicine is characterized by diversity with regard to the species of plants used. *Achyranthes aspera* Linn. is one of the numerous medicinal plant species with a remarkable therapeutic potential that is commonly recognized as Prickly Chaff flower (English). The species belongs to the Amaranthaceae family and is widely distributed as a weed throughout the tropical and subtropical regions of the world. The plant is popular in folk remedy in traditional systems of medicine in tropical Asia and African countries. Its diverse uses in the various traditional health care systems include the treatment of fever, wound healing, tooth ache, arthritis, gynecological disorders, urinary disorders, insect and snake bites, abdominal tumor, stomach pain and a number of other ailments (Raj Neeta et al., 2011). In east Africa the plant is used for treating tonsillitis, head wounds and ringworm. Different plant parts (root, stem, leaf, inflorescence, and seeds) are used individually for treating different illnesses, though the whole plant is also often used (Shendkar et al., 2011; Dangi et al., 2012).

One of the factors affecting the efficacy of medicinal plant extracts is the environment in which the plant grows. Environmental factors, to a greater extent, qualitatively and quantitatively model the chemical profiles of a plant and consequently the resulting biological activity of the extracts derived from them (Ncube et al., 2012). In addition to the pharmacological screening of plant extracts, phytochemical profiling of the extracts provides a fundamental basis for explaining the often heterogeneous activity of similar extracts collected in different seasons or growing environments.

While medical and commercial interests have always been the driving forces behind the search for new therapies, globalization is also accelerating the commodification of indigenous and local knowledge (Posey, 2002). The variable depth and extent of the ethnopharmacological bioassays used to corroborate the observed health claims of traditionally used medicinal plants ultimately contribute to evidence-based medicine. To this aim, Leonti and Casu (2013) attest that conclusive negative results may be as important as positive data. In light of this, the present study sought to investigate the antimicrobial and anthelmintic activities as well as characterisation of functional phenolic acids of *A. aspera* leaf extracts collected in different geographical locations.

## Materials and Methods

### Plant Material

Fresh leaves of *A. aspera* Linn. were collected from two different locations, i.e., (1) Ciaat (southern Zone of Eritrea 15° 03′ N; 38° 42′ E) and (2) Ukulinga (research farm at University of KwaZulu-Natal, South east of South Africa, 30° 24′ S; 29° 24′ E) during mid-April. The plants were identified at the Herbarium of UKZN and voucher specimens were deposited at the UKZN herbarium (NU).

The climatic conditions in KwaZulu-Natal, where Ukulinga is situated is sub-tropical, with temperatures influenced mostly by the warm Agulhas current that occurs along the Indian Ocean coastline. The summers (October to April) are hot and humid with temperatures averaging between 23 to 33°C and winters (May to July) are cool and (mostly) dry with temperatures averaging between16 and 25°C. Ciaat is situated in the southern zone of Eritrea, in the Horn of Africa. The hottest month is usually April to June with highs around 27–30°C with winter experienced between December and February characterized by low temperatures at night that can be near freezing point. There are two rainy seasons with light rainfall in March and April and the main rainfall from late June to the beginning of September.

### Sample Preparation

Samples from each of the above mentioned locations were separately oven dried at 50°C for 48 h. Dried plant materials were ground into powders and extracted non-sequentially (1:20 w/v) with 70% aqueous acetone (acetone aq.), water, 80% aqueous methanol and 50% aqueous methanol in an ultrasound bath for 1 h. The extracts were filtered under vacuum through Whatman’s No. 1 filter paper. The 80% aqueous methanol, 50% aqueous methanol and acetone (aq.) extracts were concentrated under vacuum using a rotary evaporator at 35°C and completely dried under a stream of air while water extracts were freeze-dried. Freshly prepared 80% aqueous methanol extracts were used in the characterisation of phenolic acids, 50% aqueous methanol extracts were used to determine protein binding affinity while the acetone (aq.) and water extracts were used in the antimicrobial and anthelmintic assays.

### Antimicrobial and Anthelmintic Activities

#### Antibacterial Activity

Minimum inhibitory concentration (MIC) values for antibacterial activity of the plant extracts were determined using the microdilution bioassay in 96-well (Greiner Bio-one GmbH, Germany) microtitre plates (Eloff, 1998) as detailed by Ndhlala et al. (2009). The pathogens used in the tests were, two Gram-negative (*Escherichia coli* ATCC 11775 and *Klebsiella pneumoniae* ATCC 13883), and two Gram-positive bacteria (*Bacillus subtilis* ATCC 6051 and *Staphylococcus aureus* ATCC 12600). Neomycin (Sigma–Aldrich) was used as positive control.

#### Antifungal Activity

The antifungal activity of the plant extracts against *Candida albicans* (ATCC 10231), a diploid fungus which exists in the form of a yeast, were evaluated using the microdilution assay (Masoko et al., 2007) as detailed by Ndhlala et al. (2009). Amphotericin B (Sigma–Aldrich) was used as positive control.

#### Anthelmintic Microdilution Bioassay

The anthelmintic activity of the plant extracts was evaluated against *Caenorhabditis elegans* var. Bristol (N2), a free living nematode, using a rapid colorimetric microdilution assay (James and Davey, 2007) with modifications to obtain minimum lethal concentration (MLC) values (Aremu et al., 2010). The assay was done twice with each sample duplicated and levamisole (Sigma–Aldrich) was used as positive control.

### Functional Phenolic Acid Quantification

The concentration of phenolic acids in ground plant material from the two locations were quantified using UHPLC-MS/MS (Waters, Milford, MA, USA) linked to a Micromass Quattro micro^TM^ API benchtop triple quadrupole mass spectrometer (Waters MS Technologies, Manchester, UK) as described by Gruz et al. (2008). For each location, three replicates of 30 mg were extracted in 1 ml of 80% aqueous methanol. Deuterium-labeled internal standards of 4-hydroxybenzoic acid (2,3,5,6-D4) and salicylic acid (3,4,5,6-D4) purchased from Cambridge Isotope Laboratories (Andover, MA, USA) were added at a final concentration of 10^-5^ mol L^-1^ to the extraction solvent prior to the homogenization. The supernatants were filtered by centrifugation (1370 × *g*, 5 min) through 0.45 μm nylon membrane filters (Micro-Spin^TM^, All-tech, Deerfield, IL, USA) and injected onto a reversed phase column (BEH C8, 1.7 μm, 2.1 × 150 mm, Waters). Gradient of acetonitrile (solvent B) and aqueous 7.5 mM HCOOH (solvent A) at a flow rate of 250 μL min^-1^ was used: 5% B for 0.8 min, 5–10% B over 0.4 min, isocratic 10% B for 0.7 min, 10–15% B over 0.5 min, isocratic 15% B for 1.3 min, 15–21% over 0.3 min, isocratic 21% B for 1.2 min, 21–27% B over 0.5 min, 27–50% B over 2.3 min, 50–100% B over 1 min, and finally 100–5% B over 0.5 min. Formic acid and acetonitrile used for preparing mobile phases were purchased from MERCK (Darmstadt, Germany). Deionized water was prepared using a Simplicity 185 system (Millipore, Bedford, MA, USA).

### Determination of Protein-precipitating Capacity of Phenolic Compounds as a Model for Wound Healing

The determination of the protein-precipitating capacity of the phenolics in the 50% aqueous methanol extracts was done according to Makkar (1999) as outlined below.

#### Formation of the Phenolic-protein Complex

To 2 ml of bovine serum albumin (BSA) solution (containing 1 mg BSA/ml acetate buffer), 50% aqueous methanol was added to the 50% aqueous methanol extract to make 3 ml (in increasing concentration of 50% aqueous methanol extract vs. 50% aqueous methanol as follows: 0.95, 0.90, 0.85, 0.80, 0.75, 0.70 ml of 50% methanol with 0.05, 0.10, 0.15, 0.20, 0.25, 0.30 ml of the MeOH extract), in triplicate in a centrifuge tube. The contents were then mixed on a vortex machine and allowed to stand at 4°C overnight in a refrigerated centrifuge. The following day (16 h), the tubes were centrifuged at 1370 × *g* for 10 min. The supernatant was carefully removed without disturbing the precipitate. To the precipitate, 1.5 ml of 1% sodium dodecyl sulfate (SDS) solution was added and mixed on a vortex machine until it dissolved. The resultant solution contained the dissolved phenolics-protein complex.

#### Determination of Phenolics in the Phenolic-protein Complex

Aliquots (1 ml) of the above dissolved Phenolic-protein complex were transferred into clean sets of test tubes. To the tubes, 3 ml of SDS-triethanolamine solution [1% SDS (w/v) and 7% (v/v) triethanolamine in distilled water] were added, followed by 1 ml ferric chloride reagent (0.01 M ferric chloride in 0.1 M HCl). Absorbance readings were taken at 510 nm after 30 min of incubation at room temperature using a UV-visible spectrophotometer. The absorbance readings were converted to gallic acid equivalents, using a standard curve. The obtained equivalents were multiplied by 1.5 to obtain the phenolics in the complex. A linear regression curve between phenolics precipitated as gallic acid equivalents and mg dry plant samples (in aliquot taken for the assay) was plotted using GraphPad Prism V6 (GraphPad Prism^®^ software Inc. La Jolla, CA, USA). The slope of the curve (mg phenolics precipitated/mg plant samples = x) represented the protein-precipitating phenolics in the sample (Makkar, 1999).

#### Protein-precipitating Capacity as a Percentage of Total Phenolics

Different aliquots (50–550 ml) of the 50% aqueous methanol extracts were made up to 1 ml with 1% of SDS, and 3 ml of the SDS-triethanolamine solution were added, followed by 1 ml of ferric chloride reagent. After incubation at room temperature for 30 min, absorbance at 510 nm was obtained as described above. A linear regression curve between phenolic acid equivalents and mg sample (in the aliquot taken) was drawn using GraphPad Prism software. The slope of the curve (mg phenolics equivalent/mg sample = y) represented the total phenolics. The protein-precipitating phenolics have already been measured as x (see section Statistical Analysis).

The percentage of total phenolics which can precipitate protein = (x/y) × 100.

#### Statistical Analysis

Data from phenolic analysis and protein-precipitating capacity of samples collected between the two locations were compared using Student *t*-test at 0.05 level of significance.

## Results

### Antibacterial, Antifungal and Anthelmintic Properties

The antibacterial, antifungal (MIC) and anthelmintic (MLC) activities of *A. aspera* leaf extracts from the two geographic locations (Ukulinga and Ciaat) are presented in **Table 1**. In this study MIC values less than 1.0 mg/ml were considered to be of notable worthy activity (good activity) and these values are highlighted in bold in **Table 1**. In this study, anthelmintic activity was tested against *C. elegans*, a free living nematode as it provides a rapid indication of anthelmintic potential of compounds/extracts. *C. elegans* remains the most frequently used test organism because of its sensitivity to most commercial anthelmintic drugs as well as the ease of culture growth and maintenance (Katiki et al., 2011; Aremu et al., 2012). It is, however, recommended that the use of at least two more organisms should be considered in order to make concrete conclusions. With the exception of water extracts against *E. coli* all leaf extracts from the specimens collected from Ciaat, Eritrea demonstrated good antibacterial, antifungal and anthelmintic activities ranging from 0.09 to 0.78 mg/ml. In contrast, only four out of the 12 extracts from samples collected from Ukulinga, South Africa exhibited good activity (MIC and MLC values between 0.59 and 0.78 mg/ml), with the rest showing weak potency of up to 6.25 against *K. pneumoniae.* On an extract to extract comparison, all the extracts from plant samples collected at Ciaat expressed significant potency compared to similar extracts from Ukulinga (**Table 1**). Notable, is the almost 9 and 17 times more potency (0.09 mg/ml) of the acetone (aq.) extracts from Ciaat compared to similar acetone (aq.) extracts from Ukulinga (0.78 and 1.56 mg/ml) against *K. pneumoniae* and *B. subtilis*, respectively. Although these were not the only extract comparisons where samples from Ciaat displayed excellent bioactivities, MIC values as low as 0.09 mg/ml, particularly against *K. pneumoniae* and *B. subtilis*, represent significant potential of this crude extract. Urinary infections and diseases of the uterus, which the plant species is claimed to cure in its traditional usage, may be caused by the tested pathogenic bacteria such as *K. pneumoniae* and *B. subtilis.* It has been largely suggested that *B. subtilis* has a low degree of virulence to humans and thus should not be used to screen medicinal plants for anti-infective activity (Van Vuuren, 2008). However, the organism was isolated from surgical wound-drainage sites, from a subphrenic abscess from a breast prosthesis and from ventriculo-atrial shunt infections (Logan, 1988). Research has also revealed its involvement in endocarditis in a drug abuse patient, fatal pneumonia and bacteraemia in leukemic patients, septicaemia in a patient with breast cancer and infection of a necrotic axillary tumor in another breast cancer patient (Logan, 1988).

**Table 1:** Antimicrobial and anthelmintic activities (MIC and MLC mg/ml) of extracts of *Achyranthes aspera* Linn. collected from two different geographical locations (Ukulinga, South Africa and Ciaat, Eretria).

Extracts	organism		MIC and MLC values (mg/ml)	
			Plant origin	
			Ukulinga, South Africa	Ciaat, Eretria
Water	Gram-negative	*Escherichia coli*	3.13	1.56
		*Klebsiella pneumoniae*	6.25	**0.78**
	Gram-positive	*Bacillus subtilis*	3.13	**0.39**
		*Staphylococcus aureus*	3.13	**0.78**
	Yeast-like and filamentous fungus	*Candida albicans*	1.56	**0.78**
	Free-living transparent nematode	*Caenorhabditis elegans*	1.17	**0.59**
Acetone (aq.)	Gram-negative	*Escherichia coli*	**0.78**	**0.39**
		*Klebsiella pneumoniae*	**0.78**	**0.09**
	Gram-positive	*Bacillus subtilis*	1.56	**0.09**
		*Staphylococcus aureus*	1.56	**0.39**
	Yeast-like and filamentous fungus	*Candida albicans*	**0.78**	**0.39**
	Free-living transparent nematode	*Caenorhabditis elegans*	**0.59**	**0.29**

*MIC, minimum inhibitory concentration, MLC, minimum lethal concentration. Acetone (aq.) = 70% aqueous acetone. The MIC values (mg/ml) for Neomycin (positive control) were: *B. subtilis* = 1.531 × 10^-*3*^; *E. coli* = 3.063 × 10^-*3*^; *S. aureus* = 6.125 × 10^-*3*^; *K. pneumoniae* = 6.125 × 10^-*3*^; Amphotericin B (positive control) for *C. albicans* was 9.77 × 10^-*3*^ and the MLC value (mg/ml) for Levamisole (positive control) against *C. elegans* was 0.042. Plant extracts with MIC values written in bold font are considered to have worth activity for further studies (MIC < 1 mg/mL).*

In addition to possible genetic polymorphism, these results could be a reflection of the existence of particular environmental condition(s) that account for the differences in the biological activity observed in this study. Ncube et al. (2012) highlight environmental factors as being the major determinants of quality of medicinal plant extracts. These being affected through either up- or down-regulation of certain specific chemical profiles within plant tissues in response to specific stimuli. It will also be of particular interest to investigate how these plants will perform if grown under the same conditions and harvested at the same age. Determination of molecular profiles of the two plants could also be of interest.

Interestingly, is that in most traditional herbal preparations, water is used as the major extractant (Hoffmann, 1989). Water extracts from samples collected from Ciaat revealed good antibacterial, anthelmintic and antifungal activity in contrast to the samples collected from Ukulinga. This represents remarkable results as the trend with most of the findings in other studies with water extracts report very poor activity (Rabe and Van Staden, 1997; McGaw et al., 2001). Moreover, the Ciaat samples demonstrated good activity against all the Gram-negative bacteria which are known to be more tolerant owing to them having an outer membrane which present a barrier to various antimicrobial molecules (Sleigh and Timbury, 1998).

### Functional Phenolic Acid Quantification

Variations in the functional phenolic acids was observed after analyzing the 80% methanolic extracts of *A. aspera* from the two locations using UHPLC-MS/MS (**Table 2**). Of the 17 phenolic acids assayed for, major variations (more than 50% differences in levels) were observed in 13 of them. This revealed major differences in the production of specific functional phenolic acids between the extracts of *A. aspera* collected from the two different locations. Rutin, chlorogenic acid (CGA) and genistein were not detected in the Ukulinga samples while they were detected at significant levels in the Ciaat samples at 1.92 ± 0.37, 0.68 ± 0.07, and 0.82 ± 0.14 μg/g DW, respectively. In another case, taxifolin was not detected in the Ciaat samples but was present at 0.22 ± 0.03 μg/g DW in Ukulinga samples.

**Table 2:** Functional phenolic acid contents in extracts of *Achyranthes aspera* Linn. collected from two different geographical locations (Ukulinga, South Africa and Ciaat, Eretria).

	Phenolic composition (μg/g DW)	
	Plant origin	
	Ukulinga, South Africa	Ciaat, Eretria
Gallic acid	1.24 ± 0.13^a^	1.08 ± 0.03^a^
Vanillic acid	3.97 ± 0.38^a^	5.69 ± 0.11^b^
Isoferulic acid	2.59 ± 0.06^a^	2.64 ± 0.08^a^
Protocatechuic acid	8.53 ± 0.24^b^	4.77 ± 0.17^a^
Syringic acid	2.24 ± 0.21^a^	4.74 ± 0.22^b^
Rutin	Not detected	1.92 ± 0.37
Salicylic acid	8.03 ± 0.19^a^	16.00 ± 1.87^b^
Gentisic acid	5.30 ± 0.28^a^	9.50 ± 1.06^b^
*p*-Coumaric acid	0.60 ± 0.10^a^	1.29 ± 0.05^b^
Trans-cinnamic acid	1.83 ± 0.34^a^	2.99 ± 0.42^b^
*p*-Hydroxybenzoic acid	9.34 ± 0.18^b^	7.76 ± 0.06^a^
Taxifolin	0.22 ± 0.03	Not detected
Chlorogenic acid	Not detected	0.68 ± 0.07
Sinapic acid	0.37 ± 0.01^a^	1.20 ± 0.06^b^
Genistein	Not detected	0.82 ± 0.14
Caffeic acid	23.76 ± 0.74^b^	8.18 ± 0.64^a^
Ferulic acid	7.76 ± 0.23^a^	8.22 ± 0.28^a^

*Different letters across each row indicate significant differences (*P* ≤ 0.05) between the levels of the phenolic acid in the extracts from the two sources as separated by the Student *t*-test (*n* = 3).*

Rutin is one of the phenolic acids found in many plants and has been shown to contributes to the antibacterial and antioxidant properties of most plant extracts (van der Watt and Pretorius, 2001; Ibtissem et al., 2012). Synthesis of rutin in plants occurs via a rutin synthase enzyme (Lucci and Mazzafera, 2009). The fact that the Ukulinga samples did not have detectable amounts of rutin could be due to the low level or no expression of rutin synthase. This may then have had an impact on the antimicrobial and other bioactivities of Ukulinga *A. aspera* samples as may be mirrored in the results obtained in this study.

Chlorogenic acid (CGA) is a natural ester of caffeic acid and (-)-quinic acid which is an important intermediate in the biosynthetic pathway of lignin (Wout et al., 2003). CGA has known antioxidant properties, inhibit tumor promoting activity (Tavani and Vecchia, 2004; Lee and Zhu, 2006) and has been shown to slow the release of glucose into the bloodstream after a meal (Johnston et al., 2003). This calls for the investigation of the antioxidants, weight loss and lowering of blood pressure of *A. aspera*. The variations in CGA in Ukulinga and Ciaat samples could affect the bioactivity of the two collection. The biosynthesis of CGA is controlled by the phenylpropanoid and the shikimic acid pathways. Given that CGA are used as precursors for the synthesis of lignin, higher activities of enzymes involved could deplete the pool of intermediates, depending on the environment and genetic polymorphism. Phenylalanine ammonia-lyase (PAL) catalyzes the first step of the general phenylpropanoide biosynthetic pathway, which produces a wide range of secondary compounds, such as flavonoides, coumarins and lignin (Koshiro et al., 2007). Koshiro et al. (2007), using semi-quantitative RT-PCR on *Coffea canephora*, it was suggested that *PAL* genes are expressed differently at different developmental stages which leads to different utilization of CGA during plant development. CGA is not directly involved in the bioactivities investigated in this study but, be that as it may, most of the phytochemical compounds do not impact bioactivity on their own or directly but work as a caucus or cartel of inter-related compounds to exert their effects.

Genistein is a phytoestrogen isoflavone that is found abundantly in the plant kingdom. Genistein has known anthelmintic, antioxidant properties and has been shown to interact with animal and human estrogen receptors. Apart from these properties, genistein has tyrosine kinase inhibitory activity mostly of the epidermal growth factor receptor (EGFR). The compound could also be responsible for wound healing properties of *A. aspera*, one of its primary uses in East African traditional medicine. As earlier mentioned and confirmed in this study (**Table 1**), *A. aspera* has anthelmintic properties against *C. elegans*, which may support its use in traditional medicine for related ailments. Genistein has been shown to exert its anthelmintic activity by inhibiting the enzymes of glycolysis and glycogenolysis (Veena et al., 2003) and disrupting the Ca^2+^ homeostasis and NO activity in the parasites (Bidyadhar et al., 2006). Genistein was found to be the active anthelmintic agent in the tuber extract of the *Felmingia vestita* which is used traditionally as an anthelmitic plant by the Khasi tribes of India (Rao and Reddy, 1991).

The absence of rutin, CGA and genistein in the Ukulinga *A. aspera* extracts might be the reason for the superior bioactivity of the Ciaat *A. aspera* extracts in the antibacterial, antifungal and anthelmintic tests (**Table 1**) as well as wound healing. However, the Ukulinga *A. aspera* extracts contained taxifolin which was not detected in the Ciaat *A. aspera* extracts (**Table 2**). Biosythesised from leucocyanidin, 2-oxoglutarate, and O_2_ by the action of leucocyanidin oxygenase, taxifolin has the capacity to stimulate fibril formation and promote stabilization of fibrillar forms of collagen. Taxifolin has potential in the cosmetics industry as, like arbutin, it inhibits cellular melanogenesis and can be used as a hypopigmenting agents in cosmetics (An et al., 2008). The ability of taxifolin to stimulate fibril formation, promote stabilization of fibrillar forms of collagen, and hypopigmention, could be important as a wound healing model of *A. aspera* (Tarahovsky et al., 2007). Taxifolin has been shown to boost the antibacterial efficacy of conventional antibiotics such as levofloxacin and ceftazidime, which supports the use of combinatory therapy for methicillin-resistant *Staphylococcus aureus* (MRSA) (An et al., 2011). This makes Ukulinga *A. aspera* extracts not inferior to Ciaat *A. aspera* extracts in terms of possible medicinal use. The quality of the plant extract in this regard becomes a subject of the complex attributes of the mixture of both qualitative and quantitative interaction of the composite chemical constituents. Environmental factors usually dictate this combinatorial chemistry in plants making the crude extracts of medicinal plants as lucrative and with limitless potential in their therapeutics properties.

### Determination of Protein-precipitating Capacity of Phenolic Compounds as a Model for Wound Healing

The results for the protein-precipitating capacity of the 50% methanolic extracts of *A. aspera* from Ukulinga and Ciaat are shown in **Table 3**. Four levels of affinity were described, with 0–20% being considered insignificant, 20–40% low, 40–70% moderate, and 70–100% high. The Ukulinga *A. aspera* extracts exhibited moderate affinity for protein binding while the Ciaat *A. aspera* extracts exhibited high affinity. It is well understood that wound healing is a complex physiological process involving several overlapping stages that could include inflammation, formation of granulation tissue, exclusion of bacterial and fungal infections as well as re-epithelialization, extracellular matrix (ECM) formation and remodeling (Perini et al., 2015).

**Table 3:** Protein-precipitating activity as a wound healing model of phenolic rich methanolic extracts of *Achyranthes aspera* Linn. collected from two different geographical locations (Ukulinga, South Africa and Ciaat, Eretria).

	Protein-precipitating activity	
	Plant origin	
	Ukulinga, South Africa	Ciaat, Eretria
Total phenolics (y)^∗^	0.129 ± 5.3 × 10^-03^	0.148 ± 0.3 × 10^-02^
Protein-precipitating phenolics (x)^∗^	0.089 ± 1.7 × 10^-02^	0.135 ± 4.4 × 10^-03^
Protein-precipitating capacity (%)	68.992 ± 0.962^a^	91.121 ± 1.372^b^

*^∗^x and y are the slopes of the curve (mg phenolics precipitated/mg plant samples) representing the protein-precipitating and the total phenolics in the sample respectively. Different letters across the protein-precipitating capacity (%) row indicate significant differences (*P* ≤ 0.05) between the protein biding capacities of the extracts from the two sources as separated by the Student *t*-test (*n* = 3).*

Phenolic compounds have been shown to enhance tissue regeneration responsible for superficial wounds and burn healing, have demonstrated antiseptic effects (antibacterial and antifungal) and have antioxidant properties (Bruneton, 1995). Phenolic-protein complexes have been implicated in wound healing. The complexes form a film which limits fluid loss and forms a physical barrier to microbial infections and forms insulations on damaged tissue protecting the wound from chemical harm. Phenolic-protein complexes also have a vaso-constructive effect on small blood vessels that limits bleeding and oozing of fluids through damaged skin or mucous membranes (Luseba et al., 2007).

The hydroxyl group in phenolic compounds is an excellent hydrogen donor that forms strong hydrogen bonds with protein carboxyl groups. To have high protein affinity, phenolic compounds must be small enough to penetrate inter-fibrillar regions of protein molecules, but large enough to crosslink peptide chains at more than one point. This then results in the above mentioned films which forms the physical barriers that aid wound healing. Apart from wound healing, extracts such as the Ciaat *A. aspera* with high protein binding affinity could exert their antimicrobial and enzyme inhibitory properties by forming hydrophobic and hydrogen bonding with the protein regions of the bacterial cell wall (Mulaudzi et al., 2012). In some cases, high protein binding affinity could transform into major negative impact on human and animal nutrition. Explanations has been put forward to describe how protein binding phenolic compounds, especially those rich in tannins influence protein utilization within the body and is generally viewed adversely. Phenolic compounds with high protein affinity bind and precipitate functional enzymes responsible for normal metabolisms thus reducing the bioavailability of nutrients and medicinal value of plants.

## Conclusion

The study investigated the antibacterial, antifungal, anthelmintic activities and characterized the functional phenolic acids as well as protein binding capacity of extracts of *A. aspera* obtained from two different geographical areas (Ukulinga, South Africa and Ciaat, Eritrea). The water and acetone (aq.) extracts of the *A. aspera* collected from Ciaat exhibited excellent antibacterial, antifungal and anthelmintic activity except the water extract against *E. coli* which had moderate activity. In contrast, the extracts of the *A. aspera* collected from Ukulinga exhibited moderate to weak activities except for the acetone (aq.) extracts which had good activity against some of the organisms tested. UHPLC-MS/MS revealed a variation in the levels of some functional phenolic compounds, with rutin, CGA and genistein being absent in the extracts from Ukulinga. Variation was also observed in the protein binding capacity, which could offer a predictive wound healing model. The extracts from Ciaat exhibited better activities in all the bioactivities assayed in the study and important functional phenolic acids were detected in these samples. The better activities portrayed by the Ciaat *A. aspera* compared to the Ukulinga one maybe be associated with several factors including the environmental conditions of the habitats, developmental stage and some genetic polymorphism.

## Author Contributions

AN, HG Conceptualized and executed the research. BN, AA, JG, MS, KD, HA helped in execution of research. AN, BN wrote the manuscript. CP, HA, JVS and all the other authors read, improved and approved the manuscript.

## Conflict of Interest Statement

The authors declare that the research was conducted in the absence of any commercial or financial relationships that could be construed as a potential conflict of interest.
